# Effect of Neurorepair for Motor Functional Recovery Enhanced by Total Saponins From *Trillium tschonoskii* Maxim. Treatment in a Rat Model of Focal Ischemia

**DOI:** 10.3389/fphar.2021.763181

**Published:** 2021-12-10

**Authors:** Le Yang, Jian-feng Lei, Jun-yao Ouyang, Man-zhong Li, Yu Zhan, Xue-feng Feng, Yun Lu, Ming-cong Li, Lei Wang, Hai-yan Zou, Hui Zhao

**Affiliations:** ^1^ School of Traditional Chinese Medicine, Capital Medical University, Beijing, China; ^2^ Beijing Key Lab of TCM Collateral Disease Theory Research, Beijing, China; ^3^ Medical Imaging Laboratory of Core Facility Center, Capital Medical University, Beijing, China

**Keywords:** total saponins from Trillium tschonoskii maxim, ischemia, neurorepair, motor functional recovery, rat model

## Abstract

*Trillium tschonoskii* Maxim. (TTM), is a perennial herb from Liliaceae, that has been widely used as a traditional Chinese medicine treating cephalgia and traumatic hemorrhage. The present work was designed to investigate whether the total saponins from *Trillium tschonoskii* Maxim. (TSTT) would promote brain remodeling and improve gait impairment in the chronic phase of ischemic stroke. A focal ischemic model of male Sprague-Dawley (SD) rats was established by permanent middle cerebral artery occlusion (MCAO). Six hours later, rats were intragastrically treated with TSTT (120, 60, and 30 mg/kg) and once daily up to day 30. The gait changes were assessed by the CatWalk-automated gait analysis system. The brain tissues injuries, cerebral perfusion and changes of axonal microstructures were detected by multimodal magnetic resonance imaging (MRI), followed by histological examinations. The axonal regeneration related signaling pathways including phosphatidylinositol 3-kinases (PI3K)/protein kinase B (AKT)/glycogen synthase kinase-3 (GSK-3)/collapsin response mediator protein-2 (CRMP-2) were measured by western blotting. TSTT treatment significantly improved gait impairment of rats. MRI analysis revealed that TSTT alleviated tissues injuries, significantly improved cerebral blood flow (CBF), enhanced microstructural integrity of axon and myelin sheath in the ipsilesional sensorimotor cortex and internal capsule. In parallel to MRI findings, TSTT preserved myelinated axons and promoted oligodendrogenesis. Specifically, TSTT interventions markedly up-regulated expression of phosphorylated GSK-3, accompanied by increased expression of phosphorylated PI3K, AKT, but reduced phosphorylated CRMP-2 expression. Taken together, our results suggested that TSTT facilitated brain remodeling. This correlated with improving CBF, encouraging reorganization of axonal microstructure, promoting oligodendrogenesis and activating PI3K/AKT/GSK-3/CRMP-2 signaling, thereby improving poststroke gait impairments.

## Introduction

Cerebral ischemia remains to be the most common cause of long-term disability worldwide (Zhou et al., 2021). Currently, recombinant tissue plasminogen activator (tPA) is the only FDA approved therapy for acute ischemic stroke. However, only approximately 5% of patients benefit from this treatment due to the narrow therapeutic time window of 4.5 h after the onset of ischemic stroke. For stroke survivors, most suffer neurological impairments (Liu and Chopp, 2016). Hence, therapeutic approaches aimed to boost rehabilitative processes during the poststroke convalescence period is of great concern (Hatakeyama et al., 2020).

*Trillium tschonoskii* Maxim (TTM) is the dried rhizomes and roots of *Liliaceae Trillium*, that has been widely used to treat cephalgia and traumatic hemorrhage in China for centuries (Li et al., 2020). Previous studies demonstrated that extracts of TTM showed remarkable effects on neuroprotection in ischemic rats (Qiu et al., 2016) and promoted restoration of impaired axons in rats subjected to spinal cord injury (Chen et al., 2015). Phytochemistry researches reported that pennogenyl and diosgenyl saponins are the principle ingredients of TTM. These two steroidal saponins have the same basic skeleton, identified as (14β,17β,25R)-Spirost-5-en aglycone and a sugar chain linked to C-3. Diosgenin is an initial substrate, and acts as a precursor to synthesize diosgenyl and pennogenyl derivatives (Tian et al., 2004; Cai et al., 2020).

More recently, we found that the n-butyl alcohol extract of TTM significantly decreased brain injuries and improved neurobehavioral function of ischemic rats during the subacute phase of stroke (poststroke day 15) (Li M. et al., 2018), suggesting the total saponins of *Trillium tschonoskii* Maxim (TSTT) may have the potential neuroprotective and repair potentiality. However, whether the TSTT leads to long-term functional recovery and brain remodeling in the chronic phase of ischemic stroke is unknown. To address this gap, we further purified the n-butyl alcohol extract of TTM by AB-8 macroporous resin, and used noninvasive MRI methodologies to investigate the possible efficacy of re-purified TSTT on the cerebral blood flow (CBF) recovery and brain tissue modifications in a rat model of focal cerebral ischemia. Motor impairment is a major cause of disability in stroke survivors (Guzik et al., 2017), so we chose the CatWalk automated gait analysis as the method to assess gait and locomotion changes in this experiment. More importantly, the differentiation of oligodendrocyte progenitor cells (OPCs) into mature oligodendrocytes leading to remyelination is one of the critical regenerative mechanisms for axonal repair, which may benefit functional recovery after brain injury. Therefore, we investigated the potential effects of TSTT on endogenous oligodendrogenesis. Specifically, the glycogen synthase kinase-3 beta (GSK-3β) has been recognized as anti-inflammatory and anti-tumor therapeutic target for steroidal saponins compounds derived from TTM (Lee et al., 2007; Li et al., 2014; Fang et al., 2016; Mou et al., 2017). Recently, the PI3K/AKT axis regulating GSK-3β and its substrate, collapsin response mediator protein 2 (CRMP-2) has been reported to regulate axonal remodeling (Lin et al., 2021). On these grounds, we further explored the axonal growth pathway (PI3K/AKT/GSK-3/CRMP-2) to gain an insight into the possible repair mechanisms underlying TSTT treatment following ischemic stroke.

## Methods

### Drugs Preparation, Animals and Experimental Design

**FIGURE 1:**
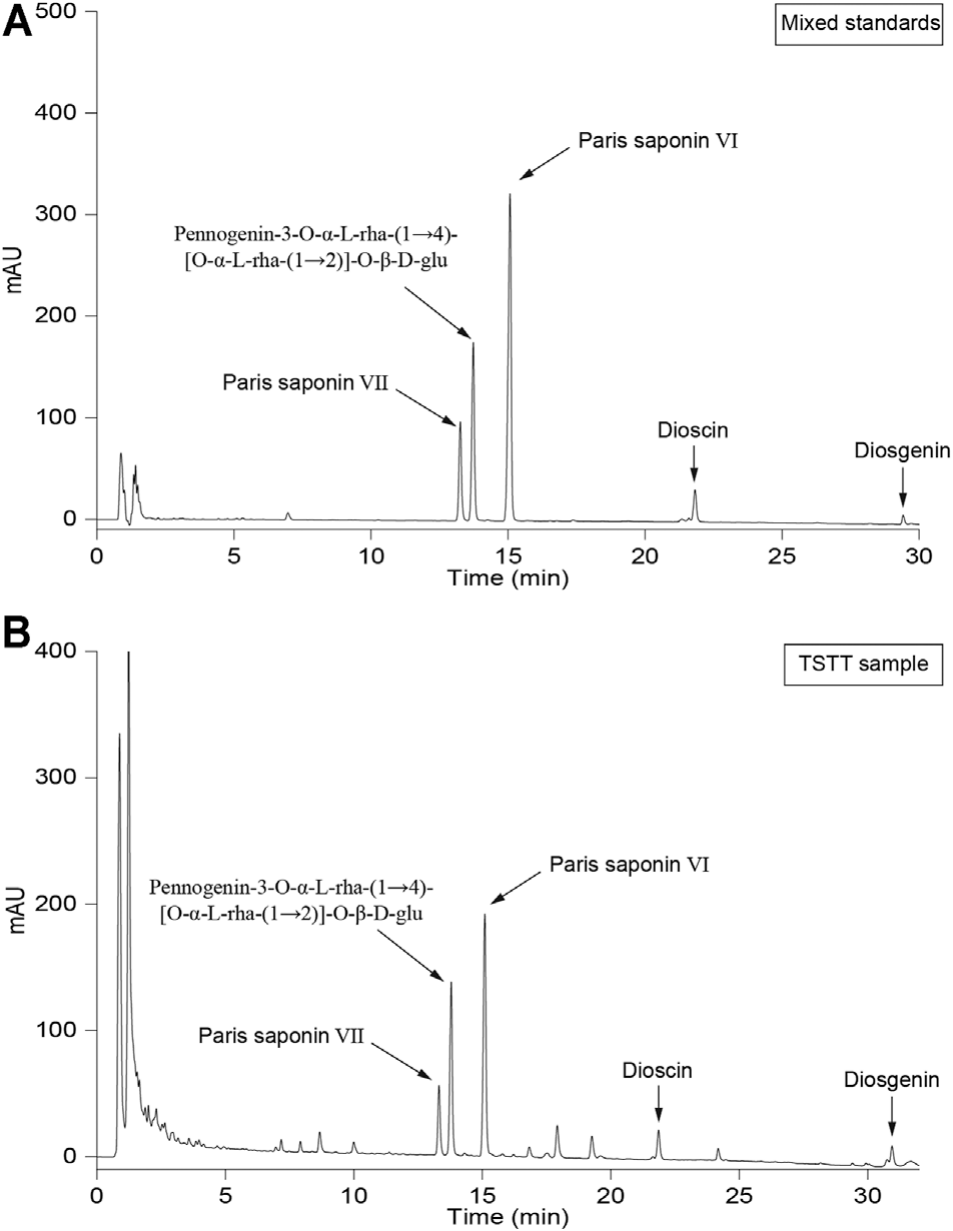
The HPLC graph of mixed standards **(A)** and TSTT **(B)**.

TSTT specimen was well-prepared in Beijing Key Lab of Traditional Chinese Medicine Collateral Disease Theory Research (Beijing, China; Batch No. 201303; Yield: 18 g/g crude drug) and stayed at - 20°C in reserve. The n-BuOH extraction (60.00 g) was subjected to AB-8 macroporous resin to elute the polysaccharide part and the fraction (purified TSTT) was yield (32.40 g; transfer rate: 87.30%). HPLC analysis showed that re-purified TSTT contained Paris saponin Ⅵ (17.54%), Pennogenin-3-O-α-L-rha-(1→4)-[O-α-L-rha-(1→2)]-O-β-D-glu (PRRG) (16.29%) and Paris saponin Ⅶ (10.05%), dioscin (3.71%) and diosgenin (1.03%) (Figure 1). TSTT dosage was calculated based on clinic dose as follows: d rat = d human × 0.71/0.11 (d human = 0.17 g/kg, d rat = 1.10 g/kg) (Lan et al., 2013). One gram of TSTT was equal to 18 g TTM crude drugs and our transfer rate of TSTT was 87.30%. So, the daily dosage of TSTT in rat should be 60 mg/kg when intragastrically administrated to rats. Thus, we selected three daily doses (120 mg/kg, 60 mg/kg, and 30 mg/kg) to investigate the effects of TSTT on brain repair and functional recovery after ischemia.

This study was designed for three series. In series 1, gait testing was performed to evaluate the roles of TSTT on movement coordination in ischemic rats. In series 2, TSTT was further to examine its effect on brain remodeling using multimodal MRI technology. In series 3, rats were sacrificed to estimate the histopathological injuries. And the axonal growth and endogenous oligodendrogenesis related molecules of PI3K/AKT/GSK-3/CRMP-2 were explored to enlighten probable mechanisms underlying subsequent recovery following TSTT treatment. Data of gait assessments, MRI examinations and histopathological observations were analyzed by researchers who blinded to the experiment design.

### Animals and MCAO Procedure

Specific-pathogen-free Sprague-Dawley (SD) male rats (Vital River Laboratory Animal Technology Co., Ltd., Beijing, China.) weighing 300–320 g, were fed three/cage with food and water ad libitum. Rats were adaptive to experimental conditions 7 days before surgery. All protocols regarding animal care were strictly complied with Animal Ethics Committee (Capital Medical University, China; No. AEEI-2018-052) and all experiments were conducted in lines with National Institute of Health for the care and use of laboratory animals.

Model of permanent cerebral ischemia (pMCAO) was performed, as described according to (Longa et al., 1989). As follows: rats were anesthetized by 3% isoflurane in a mixture of 30% O_2_/70% N_2_O. After an incision was operated on the ventral neck skin, the right common, external and internal carotid arteries (CCA, ECA, and ICA) were slightly separated from surrounding vagus nerves. The MCA occlusion (MCAO) surgery was performed using a 4-0 monofilament nylon filament (Beijing Sunbio Biotech Co. Ltd., China) introduced from the CCA via a microvascular clip maintained for the ICA. Until the filament was gently advanced to approximate 18 mm, the slipknot surrounding the distal CCA was ligated. The rats of the sham group were suffered the same process without carotid arteries ligation. Animals exhibited no obvious neurological complications were precluded from this experiment (Zhang et al., 2019b). Rats were assigned in various groups using a random number list (http:/www.99cankao.com/numbers/random-number-generator.php).

Rats with successful MCAO were randomly divided into five groups: sham, model, TSTT (120 mg/kg), TSTT (60 mg/kg) and TSTT (30 mg/kg). for the first time at post-stroke 6 h, and then once daily until day 30. TSTT was dissolved in 0.9% saline, and the sham and model group rats were fed with equivalent sodium chloride (10 ml/kg/d).

### CatWalk Test

The CatWalk test was used to quantitatively evaluate gait impairments on 30th day (*n* = 10 per group) following experimental ischemia by a DigiGait Imaging and Analysis 15.0 system (MOUSE SPECIFICS, INC., Boston, United States) (Hattori et al., 2015). Motorized device was equipped with 1.0 m length, 9 cm width transparent glass walkway and a high-speed video camera beneath the belt. Animals were adaptive to the surroundings in the glass box firstly. Then rats trained to walk on the glass walkway before running tests with the belt velocity set at 15 cm/s (Lambert et al., 2014). The light signals of paws were reflected when rats contacted the glass plate, which further were transformed into digital messages by video camera. Collected parameters in gait test were divided into two categories: 1) temporal parameters: stance time (the sustaining duration elapsed when rat paws on the belt) and swing time (the duration elapsed while rat paws did not contact the belt in the air); 2) spatial parameters: paw area (the maximum size of paws recorded when rats touched the walkway stably). The temporal parameters were recorded as milliseconds (duration) and the spatial parameters were showed as square centimeter. Behavioral detection and data analysis were executed by two observers who blind to the experimental grouping (Zeng et al., 2018).

### MRI: T2 Relaxometry Mapping, DTI Detection and ASL

Animals were anesthetized and placed in a 7.0 T Bruker animal MRI scanner (Bruker, pharma Scan, Germany) on 31st day after TSTT intervention (*n* = 8 per group).

T2-weighted imaging (T2WI) was used to examine the injuries of brains in a fast spin-echo (FSE) pulse sequence with parameters: repetition time (TR) 3,000 ms, echo time (TE) 15 ms, slice thickness 1.0 mm. The absolute T2 values were obtained from ipsilateral and contralateral sensorimotor cortex and internal capsule (Koundal et al., 2015).

Diffusion tensor imaging (DTI) was performed to evaluate the microstructures by an axial single-shot spin echo-planar imaging sequence (EPI) (TR 13760 ms, TE 21 ms, slice thickness 0.5 mm, FOV 3.5 × 3.5 cm^2^, b value 0 s/mm^2^, 1,000 s/mm^2^) (Yu et al., 2018). DTI analysis were handled by DSI Studio software (http://dsi-studio.labsolver.org/). Fractional anisotropy (FA) and radial diffusivity (RD) maps were produced. FA and RD values were analyzed in ROIs drawn on ipsilateral and contralateral SC and IC by a blinded observer.

Diffusion tensor tractography (DTT) was carried out to measure the fiber length and density of each slice of the bilateral IC using Diffusion Toolkit (version 0.6.2.2) and TrackVis software (version 0.5.2.2) (Li et al., 2014). All data were expressed as the relative value using ipsilateral values divided by the contralateral values (Liu et al., 2011).

Arterial spin labeling (ASL) was conducted to detect cerebral reperfusion (echo-planar imaging fluid-attenuated inversion recovery (EPI-FLAIR), TR 18000 ms, TE 25 ms, slice thickness 1.0 mm, FOV = 3.0 × 3.0 cm^2^) (Liu et al., 2018). The reconstruction of CBF map and heat map of ASL raw data were completed by the ParaVision 5.1 software (Bruker, Pharma Scan, Germany). ROIs were placed in the bilateral sensorimotor cortex and internal capsule on the T1 image and then duplicated to CBF map (bregma −0.2 mm) to acquire corresponding CBF metrics. The relative CBF was expressed as a ratio of the ipsilateral CBF compared to the contralateral CBF (Yang et al., 2015).

### Brain Sampling

After MRI scan, rats (*n* = 4 per group) were anesthetized and perfused transcardially with 4% paraformaldehyde. The tissues were processed as previously described (Li MZ. et al., 2018). Briefly, for transmission electron microscopy (TEM) analysis, 1 mm^3^ tissues of peri-infarct cortex were dissected and postfixed in 2.5% glutaraldehyde. After that, 2-mm-thick blocks of the brain tissue (bregma −0.8–1.2 mm) were collected, processed and embedded in paraffin. A series of coronal sections at a thickness of 4 μm were sliced from each block for histological evaluation.

### Histopathology and Immunofluorescence

Luxol Fast Blue (LFB) staining was used to evaluate damage to myelinated axons. The extent of myelin sheath injuries (rarefaction and vacuolation) was assessed by the integrated optical density (IOD) of myelinated axon. The IOD of LFB was quantified using ImageJ 6.0 software (NIH, Bethesda, Maryland 20,810, United States). Data were represented as a percentage identified by the values of ipsilateral side normalized to contralateral side, and then averaged for statistical analysis (Zheng et al., 2021).

Transmission electron microscopy (TEM) was used to examine axonal remyelination after stroke (Zhang et al., 2019c). 15 images of axons were captured in random areas of the cortex from each group and diameters of axons and myelin were measured by ImageJ. Then researchers in a blinded fashion calculated the G-ratios (axonal diameter/myelin sheath thickness) for quantitative analysis.

Double-immunofluorescent staining was adopted to evaluate the oligodendrogenesis (Zhang et al., 2010). Sections were incubated with primary antibodies at 4°C overnight as following: rabbit anti-Ki67 (a marker of newborn and proliferating cell; 1: 100, ab15580), mouse anti-neural/glial antigen 2 (NG2) (a marker for OPCs; 1: 100, ab50009), mouse anti-2*′*, 3*′*-cyclic nucleotide 3*′*-phosphodiesterase (CNPase) (a marker for mature oligodendrocytes; 1: 300, ab6319) from Abcam, Cambridge, MA, United States. After washing, sections were probed with fluorescent conjugated secondary antibodies FITC (Cat. No. 1036-02, 1: 300) and TRITC (Cat. No. 4030-03, 1:400) from SouthernBiotech, Birmingham, AL, United States. At last, sections were mounted with DAPI and coverslips (Kassis et al., 2014). The control slices were subjected to the same procedure but not incubated with the primary antibodies, for testing the specificity of antibodies. Images were captured by fluorescence microscope (Nikon, Japan) and analyzed using ImageJ software. The number of Ki67^+^, Ki67^+^/NG2^+^, and Ki67^+^/CNPase^+^ cells were counted in three random views of perilesional cortex and striatum (data presented as average number/mm^2^) (Zhang et al., 2010). The cell counting was accomplished by a researcher blind to in section code.

### Western Blotting

Western blotting analysis was conducted as previously described (Zheng et al., 2021). Proteins of each groups (*n* = 4 per group) were collected using RIPA buffer (cat. No. C1053) with phenylmethanesulfonyl fluoride (1%, cat. No. A1100) and protease inhibitor (1%, cat. No. P1260) from Applygen. The procedure was performed as a standard method of SDS-polyacrylamide gel electrophoresis (PAGE). Protein concentrations were determined by the BCA Protein Assay kit (Applygen, cat. No. P1511). Total protein (21 μg) were loaded and separated by SDS-PAGE and then transferred into PVDF membranes. The membranes was blocked with 5% BSA or skim milk for 1 h in room temperature and incubated with the primary antibodies including PI3K p85/p55 (1:3,000, GeneTex, GTX132597), PI3K p85 (1:1,000, Cell signaling Technology, 4257S), P-AKT (1:2000, Cell signaling Technology, 4060S), AKT (1:1,000, Cell signaling Technology, #4691S), Phospho-GSK-3α/β (Ser21/9) antibody (1:1,000, Cell signaling Technology, #9331), GSK-3β (1:3,000, GeneTex, GTX111192), GSK-3α (1:3,000, Abcam, ab40870), Anti-CRMP-2 (phospho T514) (1:5,000, Abcam, ab62478), Anti-CRMP-2 (1:5,000, GeneTex, GTX113420), GAPDH (1:80,000, GeneTex, GTX100118). Next day, the membranes were incubated with secondary antibody horseradish peroxidase-conjugated goat anti-rabbit IgG (1:20,000, CWBIO, CW0103S) for 1 h at room temperature and scanned by the enhanced chemiluminescence reagent (Applygen, P1050). ImageJ was used for quantitative analysis of immunoblots. Data presented as the relative values normalized to GAPDH.

### Statistical Analysis

All values were presented as the mean ± SEM. The statistics were analyzed with SPSS 21.0 (SPSS; Chicago, IL, United States). The gait results from the CatWalk were statistically analyzed using One-way ANOVA and *post hoc* LSD test for multiple comparisons (Domin et al., 2018). MRI, histological and western blot data were performed by One-way ANOVA with Bonferroni’s (Dai et al., 2020). A value of *p* < 0.05 was regarded as significant.

## Results

### TSTT Post-treatment Ameliorated Gait Impairments in MCAO Rats

**FIGURE 2:**
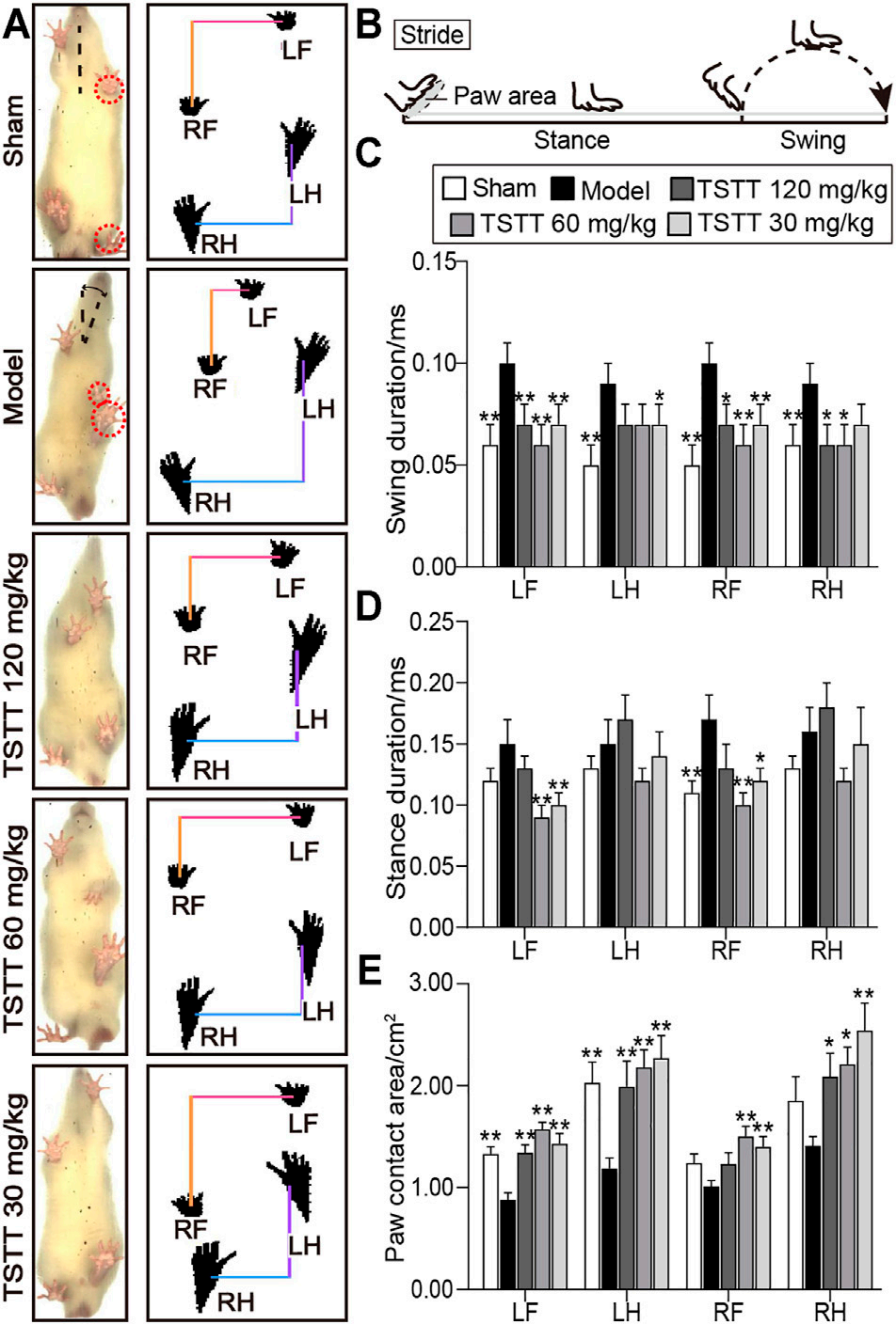
TSTT ameliorated gait impairments in the CatWalk test of rats on 30th day after pMCAO. **(A)** Gait changes of model group rats were characterized by body imbalance and limb asymmetry. **(B)** The time elapsed of one complete stride comprised of the swing duration and stance duration. Quantitative analysis of **(C)** swing duration, **(D)** stance duration and **(E)** paw contact area of LF (left-front paw), LH (left-behind paw), RF (right-front paw) and RH (right-behind paw). ^*^ *p* < 0.05, ^**^ *p* < 0.01 vs. model group.

The Catwalk-automated gait analysis test quantificationally evaluated the motor function of pMCAO rats after TSTT treatment (Figure 2A).

For the analysis of temporal parameters, the swing duration significantly increased in model group rats for all four paws as compared to sham group (*p* < 0.01, Figure 2C). TSTT (120, 60, and 30 mg/kg) significantly decreased swing duration for two front paws of MCAO rats (*p* < 0.01 or *p* < 0.05). TSTT (120, 60 mg/kg) reduced swing duration of right-behind paw (*p* < 0.05) and TSTT (30 mg/kg) showed a decline in left-behind paw compared to model rats (*p* < 0.05).

In addition, the stance duration demonstrated a significant increase in right-front paw of model group contrast to the sham group (*p* < 0.01, Figure 2D). TSTT (60, 30 mg/kg) treatment dramatically reduced the stance duration of two front paws compared to the model group (*p* < 0.01 or *p* < 0.05).

For the evaluation of spatial parameter, the paw contact area prominently decreased in two contralateral paws of the ischemic rats as compared to sham-operated rats (*p* < 0.01, Figure 2E). The contact area in ipsilateral paws was also descending observed in the model group. In contrast, the contact area of left-sided paws and right-behind paws was apparently increased in TSTT (120, 60, and 30 mg/kg) treatment group rats compared to model group rats (*p* < 0.01 or *p* < 0.05). Moreover, TSTT (60, 30 mg/kg) also significantly increased the paw contact area of right-front paw (*p* < 0.01). Our data indicated that TSTT treatment alleviated the gait deficit of ischemic stroke rats.

### TSTT Post-treatment Alleviated Brain Tissues Injuries in MCAO Rats

**FIGURE 3:**
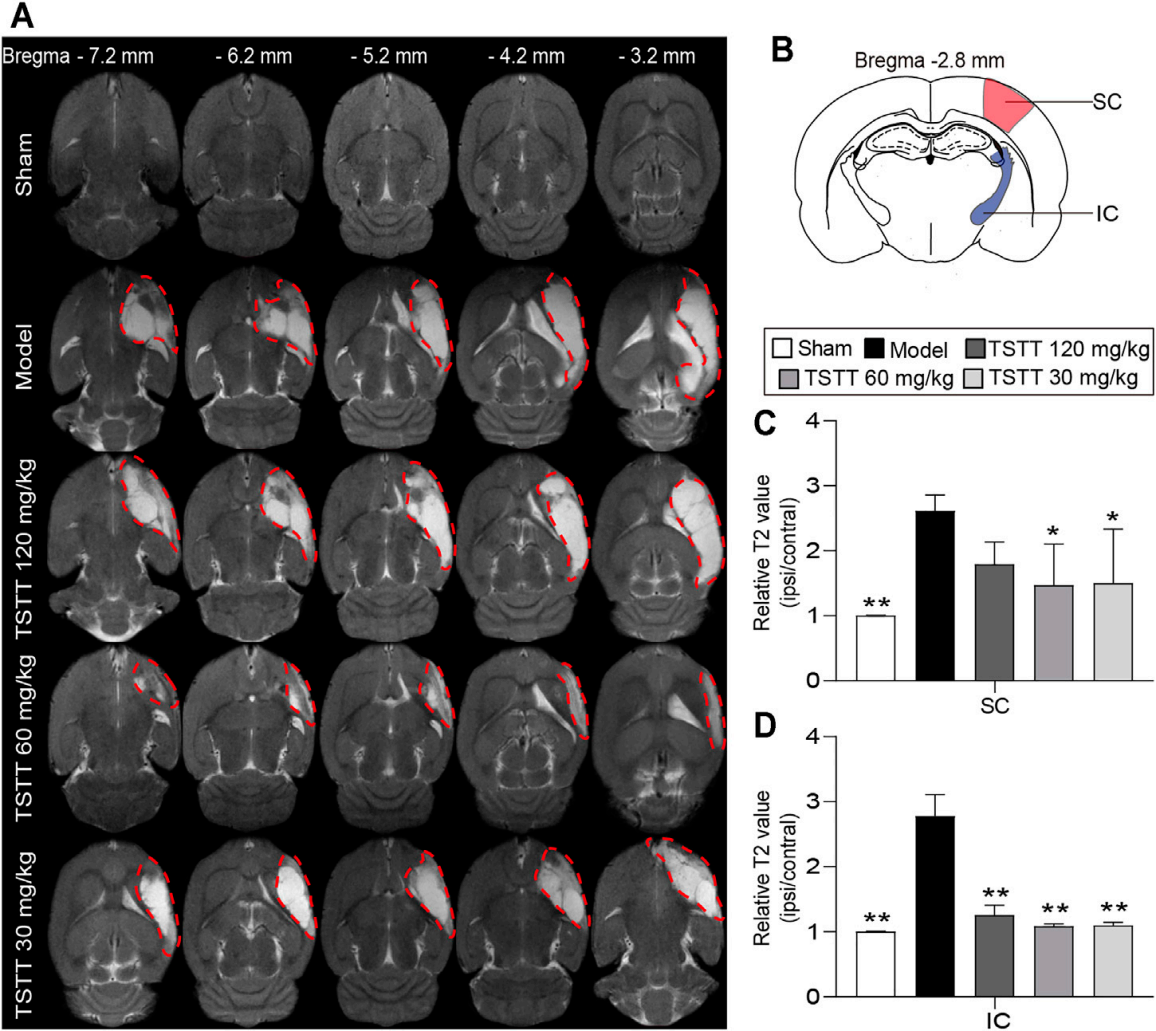
TSTT alleviated brain tissues injuries of rats on 31st day after pMCAO. **(A)** Representative axial T2WI images obtained from sham, model, TSTT (120, 60, and 30 mg/kg) group rats from bregma −7.2 mm to −3.2 mm (Abnormal T2 hyperintensities were depicted by red dashed). **(B)** Schematic illustration of sensorimotor cortex (SC, red) and internal capsule (IC, blue) of rat brain diagram. The relative T2 relaxometry values in **(C)** SC and **(D)** IC were automatically collected, respectively. ^**^ *p* < 0.01, ^*^ *p* < 0.05 vs. model group.

T2WI was utilized to determine the brain tissues damages. Axial T2WI maps showed abnormal hyperintensities in the ipsilateral MCA territory after ischemia (Figure 3A). The relative T2 relaxation times in the sensorimotor cortex and internal capsule of the model group rats were higher than that of the sham group (*p* < 0.01, Figures 3C,D), whereas in the TSTT (60, 30 mg/kg) groups, these parameters were significantly decreased in the corresponding brain regions (*p* < 0.01 or *p* < 0.05). Notably, poststroke TSTT 120 mg/kg significantly reduced the relative T2 times in the internal capsule compared to the model group (*p* < 0.01).

### TSTT Post-treatment Preserved the Microstructure of Axon and Myelin Sheath in MCAO Rats Based on MRI Parameters

**FIGURE 4:**
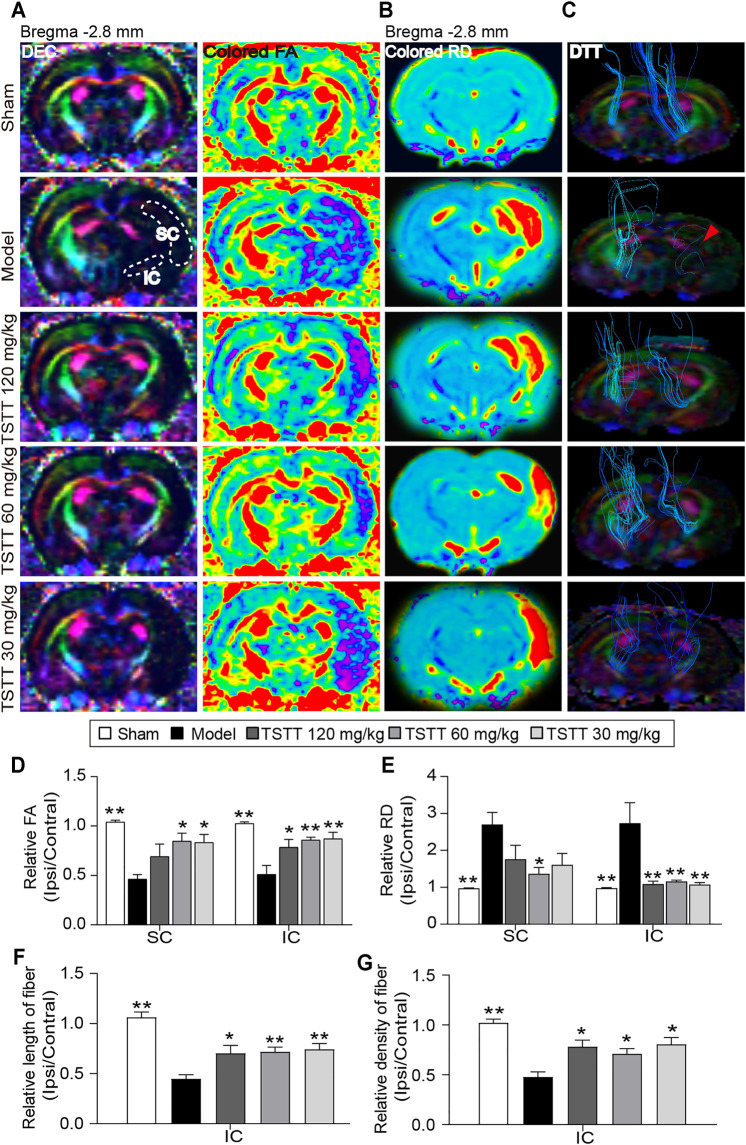
TSTT preserved microstructure of axon and myelin sheath of rats on 31st days after pMCAO. **(A)** Representative directional encoded color (DEC) and colored fractional anisotropy (FA) were obtained from sham model, TSTT (120, 60, and 30 mg/kg) group (White dash indicated the sensorimotor cortex (SC) and internal capsule (IC) regions in the left column). **(B)** Typical radial diffusivity (RD) maps (bregma −2.8 mm). **(C)** Reconstructed diffusion tensor tractography (DTT) images (Red arrowhead indicated ruptured and sparse fibers of the model group rats). Quantitative analysis of **(D)** rFA and **(E)** rRD values in the SC and IC of each group rats. The relative **(F)** length and **(G)** density of fibers in IC were calculated. ^*^ *p* < 0.05, ^**^ *p* < 0.01 vs. model group.

DTI was used to measure the microstructural integrity changes of axon and myelin sheath after MCAO. The FA value obtained with DTI sequence characterizes the integrity of axonal microstructure (Figure 4A). Using the FA measurements, quantitative data demonstrated that the relative FA in the ipsilateral sensorimotor cortex and internal capsule of model rats were significantly lower than that of the sham group (*p* < 0.01, Figure 4D). However, the rFA of the ipsilateral sensorimotor cortex and internal capsule was significantly higher in the TSTT (60, 30 mg/kg) treated group (*p* < 0.01 or *p* < 0.05). Especially, TSTT 120 mg/kg treatment obviously increased rFA in the ipsilateral internal capsule (*p* < 0.05).

In addition, DTI-derived parameters RD, a marker of the alterations of myelin sheath, showed that the ratio of RD value (ipsilateral/contralateral) of the model group was significantly increased as compared to the sham group (*p* < 0.01, Figure 4B). While, TSTT (120, 60, and 30 mg/kg) treatments significantly reduced the rRD values contrast to the model group in the internal capsule (*p* < 0.01, Figure 4E). It’s notable that rats treated with TSTT 60 mg/kg also significantly decreased rRD in the ipsilateral sensorimotor cortex as compared to model groups (*p* < 0.05). These DTI observations indicated that treatment with TSTT improved microstructural integrity of axon and myelin.

Next, DTT was performed to observe the integrity and connectivity of axonal fibers, which represented reorganization of the internal capsule (Figure 4C). Quantitative analysis demonstrated that the relative length and density of axonal fibers of the model group were significantly decreased compared to those of the sham group in the internal capsule (*p* < 0.01, Figures 4F,G). However, the relative length and density of axonal fibers were markedly elevated by TSTT (120, 60, and 30 mg/kg) treatments contrast to the model group (*p* < 0.01 or *p* < 0.05). These results suggested that TSTT might contribute to the axonal remodeling after ischemia.

### TSTT Post-treatment Preserved Myelinated Axons and Ultrastructure of Axonal Fibers in MCAO Rats

**FIGURE 5:**
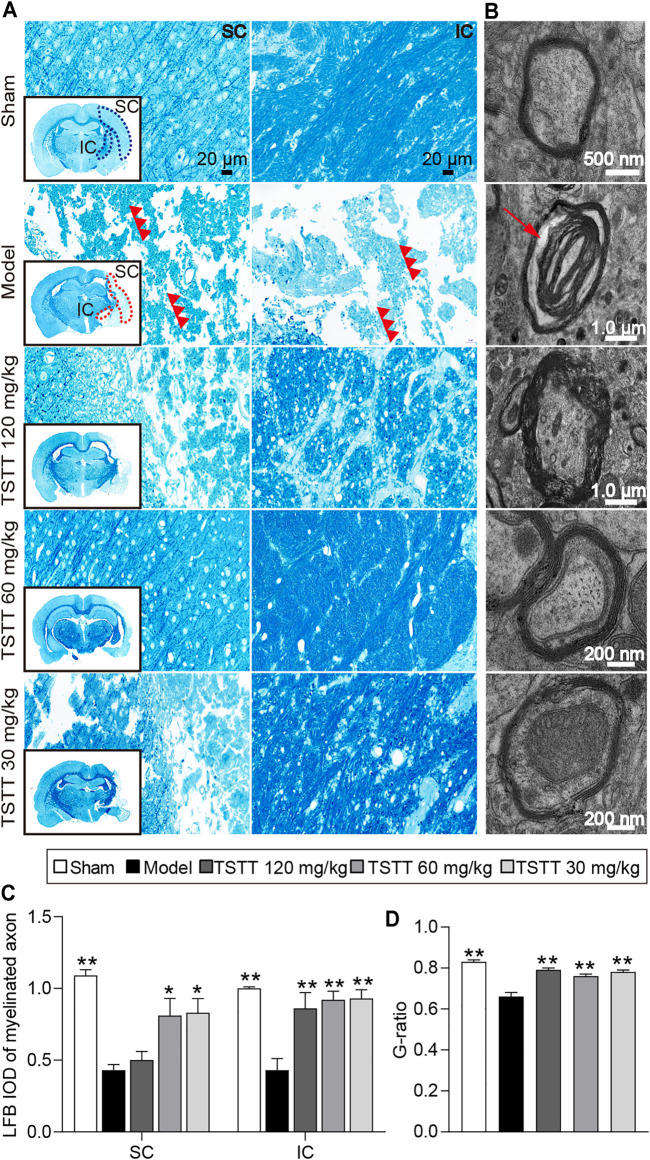
TSTT preserved myelinated axons and myelinated axons of rats on 31st day after pMCAO. Photomicrographs of tissues obtained in the brain sensorimotor cortex (SC) and internal capsule (IC). **(A)** LFB staining characteristic images in bregma −2.8 mm are displayed (black box) and magnified in surroundings (Scale bar = 20 μm, 40 × magnification). The vacuolation and fragments of myelinated axonal fiber were pointed out by red triple-arrowheads. **(B)** Representative TEM images for ultrastructure of axons and myelin sheath (Red arrow indicated myelin sheath splitting from axons). Quantitative analysis of **(C)** LFB IOD of myelinated axon in SC, IC (*n* = 4) and **(D)** the G-ratio (*n* = 2). ^*^ *p* < 0.05, ^**^ *p* < 0.01 vs. model group.

LFB staining was employed to evaluate the myelination of axonal fibers. The present results showed the myelinated axons had complete structures and arranged trimly in sham group rats, while the loss of axon and myelin in the ipsilateral hemisphere were detected in rats after stroke (Figure 5A). Quantitative analysis demonstrated that the LFB IOD of myelinated axons in the model group were decreased in the sensorimotor cortex and internal capsule as compared to the sham group (*p* < 0.01, Figure 5C). After TSTT (60, 30 mg/kg) treatment, the LFB IOD of myelinated axons in the peri-infarct sensorimotor cortex and internal capsule were higher than that of the model group (*p* < 0.01 or *p* < 0.05). Notably, TSTT 120 mg/kg also markedly elevated the LFB IOD of myelinated axons in the internal capsule compared with the model group (*p* < 0.01).

Further, TEM was used to detect the ultrastructural injuries of axons and myelin sheath. In the sham group, the axons were wrapped with myelin tightly, and lamellar structures of myelin sheath were complete. After MCAO, myelin was split from axons and loosen (Figure 5B). Quantitation showed that significant decreases in the g-ratio were observed in the model group rats compared to the sham group (*p* < 0.01, Figure 5D), indicating a loosen in myelin. TSTT (120, 60, and 30 mg/kg) treatment dramatically boosted the g-ratio in comparison of the model group (*p* < 0.01). Our results suggested that TSTT might preserve axonal myelination and accelerate axonal remyelination after stroke.

### TSTT Post-treatment Improved Cerebral Blood Flow That Correlated With Improved Tissues Injuries of Sensorimotor Cortex and Internal Capsule in MCAO Rats

**FIGURE 6:**
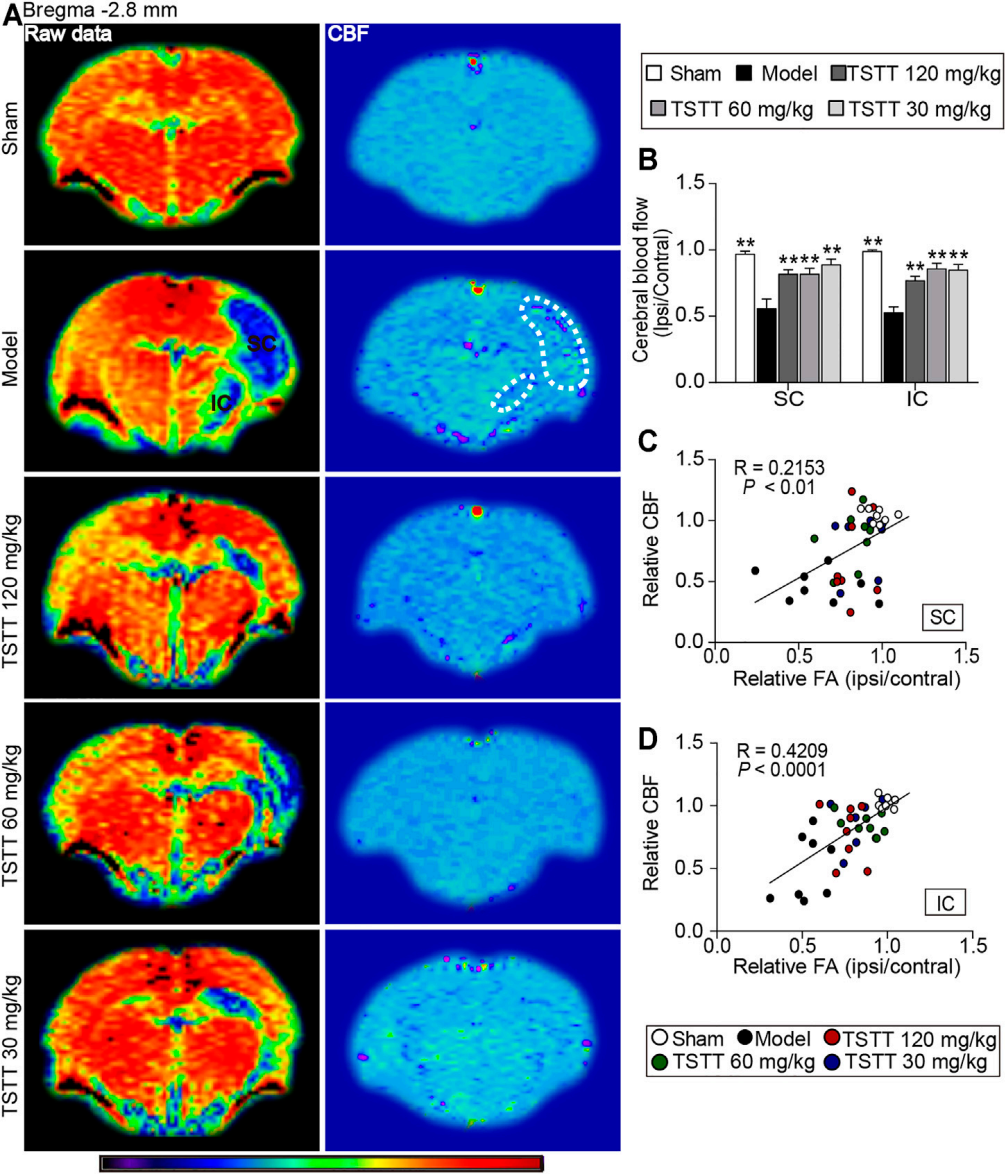
TSTT improved cerebral blood flow that correlated with improved tissues injuries of sensorimotor cortex and internal capsule of rats on 31st days after pMCAO. **(A)** Representative ASL raw data maps (left column) and CBF maps (right column) were obtained from each group. Cerebral blood flow of sensorimotor cortex (SC) and internal capsule (IC) were decreased (Blue territory in the left column, white dotted line of corresponding regions in the right column). **(B)** Quantitative analysis of the relative CBF in the ipsilateral and contralateral SC and IC. Linear correlation between relative CBF with relative FA in the **(C)** SC and **(D)** IC (*n* = 8). ^**^ *p* < 0.01 vs. model group.

CBF perfusion was assessed using ASL technique (Figure 6A). Quantitative data demonstrated that the relative CBF of ipsilateral sensorimotor cortex and internal capsule in the model group were significantly decreased when compared to the sham group (*p* < 0.01, Figure 6B). Notably, TSTT (120, 60, and 30 mg/kg) markedly increased the rCBF in the corresponding brain regions than that in the model group (*p* < 0.01).

Next, Pearson linear regression was analyzed to investigate the correlation between the rCBF and the corresponding rFA values. The analysis results showed that the rCBF had significantly positive correlation with the rFA values in the sensorimotor cortex (*R* = 0.2153, *p* < 0.01) and internal capsule (*R* = 0.4209, *p* < 0.0001), suggesting the CBF restoration may potentially promote axonal remodeling of the ipsilateral sensorimotor cortex and internal capsule after ischemia (Figures 6C,D).

### TSTT Post-treatment Promoted Perilesional Oligodendrogenesis in MCAO Rats

**FIGURE 7:**
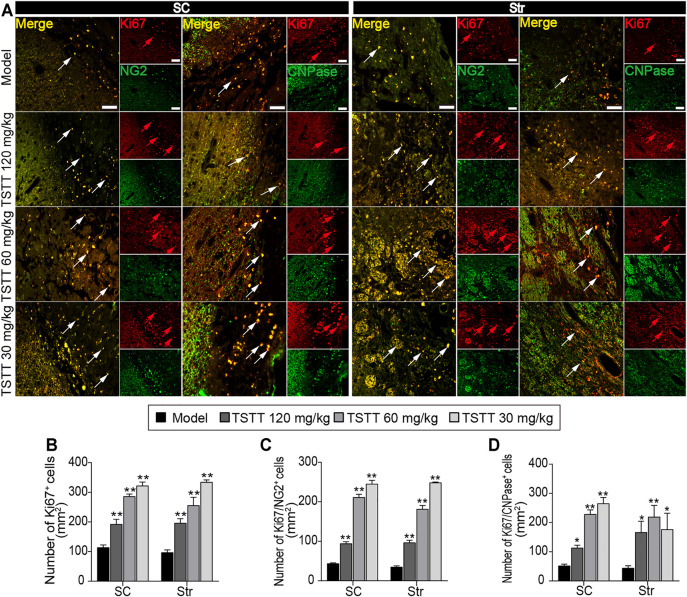
TSTT promoted perilesional oligodendrogenesis of rats on 31st days after pMCAO. **(A)** Typical immunofluorescent images of Ki67^+^ with NG2^+^ and CNPase^+^ were captured from the perilesional sensorimotor cortex (SC) and striatum (Str) (red arrowhead indicated Ki67^+^ cells, white arrowhead indicated co-localized cells). Quantitation of the number of **(B)** Ki67^+^, **(C)** Ki67^+^/NG2^+^, and **(D)** Ki67^+^/CNPase^+^ cells per mm^2^ in the perilesional SC and Str. Scale bar = 50 μm (*n* = 4). ^*^ *p* < 0.05, ^**^ *p* < 0.01 vs. model group.

Double immunofluorescent staining was performed to detected oligodendrogenesis after ischemia. First, the proliferation of OPCs was detected by Ki67/NG2^+^ immunofluorescence. As NG2 is widely expressed in OPCs, stroke irritated Ki67/NG2^+^positive cells in perilesional sensorimotor cortex and striatum (Figure 7A). TSTT (120, 60, and 30 mg/kg) increased the number of Ki67-positive cells and further boosted the number of Ki67/NG2^+^ cells in the peri-infarct striatum and cortex compared to the model group (*p* < 0.01), suggesting that TSTT promoted the newly-born OPCs proliferating (Figures 7B,C). Then, double-stained Ki67/CNPase^+^ was examined for the OPCs maturation (Figure 7A). Statistics showed that the number of Ki67/CNPase^+^ cells in the TSTT (120, 60, and 30 mg/kg) treated group were much more than that of the model group (*p* < 0.01 or *p* < 0.05), indicating that TSTT facilitated the OPCs to differentiate into the mature oligodendrocytes (Figure 7D). These overall data elucidated that TSTT is important in promoting axonal remyelination after ischemia.

### TSTT Post-treatment Activated the PI3K/Akt/GSK-3/CRMP-2 Pathway in MCAO Rats

**FIGURE 8:**
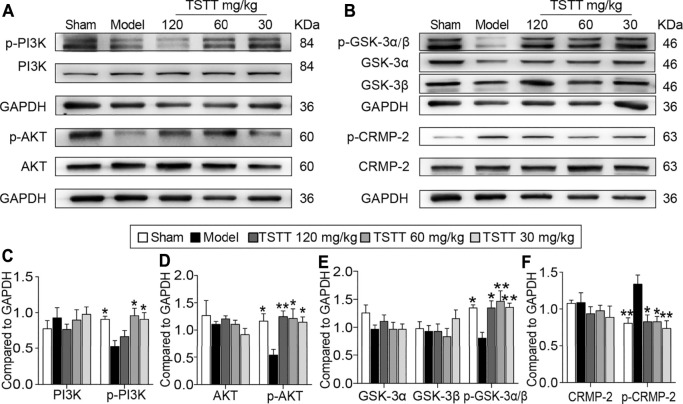
TSTT activated the PI3K/Akt/GSK-3/CRMP-2 pathway in rats on 31st day after pMCAO. **(A)** The representative bands of p-PI3K (Tyr 467/Tyr 199), PI3K, *p*-AKT (Ser 473) and AKT. **(B)** Typical protein blot of pGSK-3α/β (Ser 21/9), GSK-3α, GSK-3β, *p*-CRMP2 (Thr 514) and CRMP2. Quantitative analysis for protein expressions of **(C)** p-PI3K and PI3K, **(D)** *p*-AKT and AKT, **(E)** pGSK-3α/β, GSK-3α and GSK-3β, **(F)** *p*-CRMP-2 and CRMP-2 (*n* = 4). ^*^ *p* < 0.05, ^**^ *p* < 0.01 *vs.* model group.

Because the activation of GSK-3β is implicated in the therapeutic target for steroid saponins compounds derived from TTM, we first investigated the effects of TSTT on GSK-3 activation (Figure 8B). Compared with the sham group, the expression of the inactive form of GSK-3α/β (Ser 21/9) was significantly decreased in the model group (*p* < 0.05), with no significant difference in the total protein levels of GSK-3α and GSK-3β among all groups. Compared with MCAO group, the expressions of *p*-GSK-3α/β were markedly upregulated by TSTT (120, 60, and 30 mg/kg) treatments (*p* < 0.01 or *p* < 0.05, Figure 8E). These results suggested that TSTT effectively prevented GSK-3 activation after ischemic stroke.

As GSK-3β is associated with regulating axonal microtubule through phosphorylating downstream targets CRMP-2, we quantified both *p*-CRMP-2 and the total CRMP-2 protein levels (Figure 8B). Western blotting analysis showed that MCAO induced a profound elevation of CRMP-2 phosphorylation compared to the sham group rats (*p* < 0.01), but TSTT (120, 60, and 30 mg/kg) decreased the phosphorylation of CRMP-2 compared with the model group (*p* < 0.01 or *p* < 0.05, Figure 8F). However, total CRMP-2 remained unchanged (*p* > 0.05).

PI3K/AKT is the upstream signaling of GSK-3β. Further, we determined whether PI3K/AKT is involved in TSTT-mediated axonal regeneration after stroke (Figure 8A). The results showed that the active form of PI3K/AKT, p-PI3K (Tyr 467/Tyr 199) and *p*-AKT (Ser 473) were significantly decreased in MCAO model group as compared to the sham group (*p* < 0.05), but TSTT (60, 30 mg/kg) upregulated the expression of p-PI3K and *p*-AKT (*p* < 0.05), and TSTT 120 mg/kg also increased the expression of *p*-AKT in comparison with MCAO group (*p* < 0.01, Figures 8C,D).

## Discussions

In the present study, our data demonstrated that TSTT improved long-term neurological recovery as evaluated by automatically quantitative gait analysis tests up to 30 days after ischemia. Furthermore, our data indicated that TSTT played an essential role in axonal remodeling by improving CBF and stimulating endogenous oligodendrogenesis in ischemic cerebral tissues after stroke. Taken together, these data supported that TSTT is a potential therapeutic candidate for neurorestorative treatment after ischemic stroke.

It is well known that ischemic stroke not only causes gray matter damages but elicits subcortical white matter damages, as characterized by demyelination and axonal injuries (Zatorre et al., 2012). MRI, as a noninvasive imaging modality, is sensitive to tissue damages irritated by stroke (Saar and Koretsky, 2019). Currently, T2WI revealed that hyperintensities in the MCA territory of the ischemic side, whereas the relative T2 values of the sensorimotor cortex and internal capsule were dramatically lowered after TSTT treatment. Moreover, MRI-based DTI is conducted to noninvasively detect the microstructural changes following ischemic stroke (Van Tilborg et al., 2017). In the present study, DTI images showed that decreased FA values in the sensorimotor cortex and internal capsule of the ischemic hemisphere after ischemia, while TSTT treatment significantly elevated FA value in the corresponding areas. In general, the decreased FA value is possibly attributed to structural breakdown of axons, but enhanced FA represents axonal remodeling after stroke (Son et al., 2021)*.* Data herein indicated that TSTT might facilitate microstructural integrity after ischemic stroke. In correspondence with DTI results, the DTT maps revealed that TSTT intervention significantly elevated fiber density and fiber length in the internal capsule, indicating axonal fiber restoration. Furthermore, LFB staining and electron microscope analysis provided direct evidence showing that TSTT could restore axonal and myelin microstructural integrity. Collectively, this study strongly supported the beneficial effects of TSTT in relieving cerebral tissue damages and facilitating axonal remodeling, predominantly in the ipsilesional sensorimotor cortex and internal capsule following ischemic stroke.

Internal capsule constitutes the core of the subcortical motor pathway. Among white-matter lesions, internal capsule infarct is known to lead to long-term disabilities (Kim et al., 2014). Thus, TSTT protected both sensorimotor cortex and internal capsule against ischemic stroke, indicating that TSTT are expected to improve neurological outcomes. Based on this, we quantitatively investigated the therapeutic effect of TSTT treatment on gait impairment using the CatWalk-assisted automated gait analysis system. Gait testing is a favorable tool quantitatively used to evaluate motor disabilities after ischemic stroke (Guzik et al., 2017). In this study, gait data showed that a prominent gait dysfunction in the rats following MCAO, including significantly increased swing duration and stance duration, and reduced paw contact area. The temporo-spatial parameters of gait changes are the most common deficits among poststroke disabilities. In particular, increased stride time (swing duration and stance duration) is robustly associated with a hemiplegic and asymmetric gait performance, suggesting that stroke reduced limb coordination. And the reduced contact area of paws was attributed to reduced weight bearing capacity of the limbs (Domin et al., 2018). In contrast, TSTT decreased temporal parameters, and increased the paw contact area compared to the model group. With the information obtained from CatWalk-automated gait analysis, we demonstrated that TSTT improved gait function in the sequelae phase of ischemic stroke.

Improving a constant cerebral perfusion is important strategies for brain remodeling following ischemic stroke (Stamatovic et al., 2019). ASL perfusion imaging is a useful tool for assessing dynamic cerebral blood changes (Aracki-Trenkic et al., 2020). In the present study, ASL perfusion imaging indicated poor collateral status in the ipsilateral ischemic region of the brain following MCA occlusion. In contrast, TSTT treated rats exhibited augmented CBF in the ischemic sensorimotor cortex and internal capsule compared to the MCAO rats. Especially we analyzed the correlation between CBF and DTI indices, and noticed that higher CBF value corresponded to increased FA in the ischemic regions following ischemic stroke. The data herein confirmed that improved regional CBF played a critical role in axonal remodeling after TSTT treatment.

In response to ischemia, endogenous oligodendrocyte precursor cells proliferating and subsequently maturing into oligodendrocytes could be activated (Ramos-Cejudo et al., 2015). It should be noted that endogenous oligodendrogenesis is not sufficient for the remodeling of myelin sheath (Shahsavani et al., 2021). Amplified cerebral blood flow along the ischemic territory might provide a favorable environment for oligodendrogenesis (Li Y. et al., 2021). In this study, we found that the numbers of NG2^+^-OPCs and newly generated CNPase^+^-oligodendrocytes around the peri-infarct regions were enhanced following TSTT treatment, suggesting that TSTT could boost oligodendrogenesis. Further, using DTI-derived parameters RD value characterized as the alterations of myelin sheath, we found that TSTT treated rats showed reduced RD in the ipsilateral sensorimotor cortex and internal capsule. It is currently believed that the phenomenon of decreased RD is associated with remyelination (Zhang et al., 2019c). Thus, the present study suggested that TSTT-induced oligodendrogenesis might help to myelin formation and thereby promoted axonal remodeling during the late recovery phase following ischemic stroke. However, detailed mechanisms by which TSTT played its pivotal roles in this process remains to be elucidated.

Multiple findings indicated that saponins compounds derived from TTM possess potential properties of anti-inflammation and anti-tumor mediated by GSK-3β signals. For example, it is documented that Paris saponin VII suppressed colorectal cancer cells growth through GSK-3β associated Ras signaling pathway (Li et al., 2014). Paris saponin VII impeded ovarian cancer cell migration and invasion by regulating the GSK-3β pathway (Mou et al., 2017). Diosgenin conferred resistance to the melanogenesis associated with regulating of GSK-3β expression by activation PI3K signaling (Lee et al., 2007). In addition to the pivotal roles in the inflammatory injuries and tumor formation, GSK-3 kinases and its upstream or downstream also play essential roles in axonal remodeling during ischemia (Darshit and Ramanathan, 2016). Especially, GSK-3β and its important downstream effector CRMP-2 are regarded as playing a pivotal role in axonal growth (Lin et al., 2021). Thus, we analyzed the GSK-3β/CRMP-2 regulatory pathways, and results clearly showed that TSTT boosted the phospho-GSK-3β expression accompanied by decreased phosphorylation of CRMP-2 after ischemia. CRMP-2 as a microtubule binding protein, serves a crucial role in microtubule dynamics and neurite outgrowth. GSK-3β blocks the binding activity of CRMP-2 to tubulin by phosphorylating CRMP-2, leading to microtubule destabilization and growth cone collapse (Gim et al., 2013).

As the upstream signaling of GSK-3β, PI3K/AKT is required for axon formation and extension after injury (Xiong et al., 2012). Based on this, we further detected the expression of phosphorylated PI3K and its downstream target AKT, and found that treatment with TSTT significantly induced PI3K/AKT activation, which might inactivate GSK-3β. Collectively, the present results showed that TSTT-induced axonal remodeling, at least in part, via activating PI3K/AKT followed by inactivated GSK-3β and attenuated phosphorylation of CRMP-2 after ischemic stroke. The enzymatic activity of GSK-3 is inhibited by serine phosphorylation in either of the two isoforms of GSK-3, serine-9 in GSK-3β or serine 21 in GSK-3α. It must be pointed out that TSTT also up-regulated phosphorylation of GSK-3α at Ser 21. This result promoted us to hypothesize that GSK-3α might potentially be involved in the TSTT-inducted axonal remodeling after ischemia, which deserves future investigation.

It’s worth noting that TSTT are mainly constituted by two kinds of steroidal saponins, pennogenin, and diosgenin saponins, which possess different biological and pharmacological properties (Hernandez-Vazquez et al., 2020; Ahmad et al., 2021). Specifically, pennogenin saponins have anti-inflammatory, anti-tumor and anti-hemorrhagic activity (Wang et al., 2013). For example, Paris saponin VII showed robust effect of anti-inflammation in LPS-stimulated RAW264.7 cells by reducing the levels of pro-inflammatory cytokines and enzymes, simultaneously inhibited the inflammation response to ear edema and granuloma formation in mice (Zhang C. et al., 2019). Notably, diosgenin saponins exerted neuroprotection against neuroinflammation (Wang et al., 2017). It was reported that diosgenin, the main metabolite of diosgenin glycosides, could improve cognitive function and promote neurite outgrowth in normal mice (Yang et al., 2021). Specifically, diosgenin exhibited neuroprotective role in a hyperglycemia-induced cerebral ischemia zebrafish model by reducing oxidative damage and inflammation (Oyelaja-Akinsipo et al., 2020). In addition, previous study showed that dioscin and diosgenin treatments alleviated cerebral ischemia reperfusion injury in rats and reduced the levels of multiple pro-inflammatory cytokines (Li et al., 2021a). Based on these results, we could confirm that TSTT promoted repair of axonal injury and motor functional recovery following ischemic stroke, may be due to these two kinds of steroidal saponins.

The major limitations of this study should be noted. Firstly, the pharmacologic effects of pennogenin saponins and diosgenin saponins from TSTT needed to be further investigated. Secondly, the current findings indicated that GSK-3 signal transduction cascades might be linked with TSTT induced axonal regeneration after stroke, however, the specific mechanisms remain unclear. Thirdly, the present study was conducted to clarify the role of TSTT on post-stroke brain repair investigated by MRI in rats on 30 days after ischemic stroke, but very little is known about the time course of the axonal remodeling under TSTT long-term treatment. Future investigations that designed to perform longitudinal MRI study might be useful for better understanding of TSTT-induced brain restoration following ischemic stroke.

Although further experimental studies are needed, with the information obtained from MRI and histologic evaluation, our present study suggested that TSTT treatment could promote long-lasting rehabilitation following ischemic stroke, as manifested by attenuating brain tissues injuries, promoting axonal remodeling accompanied with CBF restoration, boosting endogenous oligodendrogenesis, which might at least partially through activating PI3K/AKT/GSK-3β/CRMP-2 signaling pathway after ischemic stroke, all of which was pivotal for the neural repair following ischemia. These results provided a valuable basis for further investigation of detailed cellular and molecular mechanisms with regard to TSTT activated restoration after ischemic stroke.

## Data Availability

The raw data supporting the conclusion of this article will be made available by the authors, without undue reservation.
